# Comparison of three strains of diabetic rats with respect to the rate at which retinopathy and tactile allodynia develop

**Published:** 2010-08-15

**Authors:** T.S. Kern, C.M. Miller, J. Tang, Y. Du, S.L. Ball, L. Berti-Matera

**Affiliations:** 1 Case Western Reserve University, Center for Diabetes Research, Cleveland, OH; 2 VAMC Research Service 151, Cleveland, OH

## Abstract

**Purpose:**

We compared three rat strains to determine if different strains develop early-stage diabetic retinopathy or sensory neuropathy at different rates.

**Methods:**

Sprague Dawley, Lewis, and Wistar rats were made diabetic with streptozotocin. Diabetic and nondiabetic animals had retinal vascular pathology measured at eight months of diabetes. The number of cells in the retinal ganglion cell layer (GCL), retinal function (using electroretinography [ERG]), and retinal levels of inducible nitric oxide synthase (iNOS), cyclooxygenase2 (COX2), and vascular endothelial growth factor (VEGF) were measured at four months of diabetes. Tactile allodynia was assessed in hind paws at two months of diabetes.

**Results:**

Diabetes of eight months’ duration resulted in a significant increase in retinal degenerate capillaries and pericyte ghosts in Lewis and Wistar rats, but not in Sprague Dawley rats. A significant loss of cells in the GCL occurred only in diabetic Lewis rats, whereas Wistar and Sprague Dawley rats showed little change. Diabetes-induced iNOS and VEGF were statistically significant in all strains. Cyclooxygenase 2 (COX2) was significantly elevated in the Sprague Dawley and Wistar strains. Lewis rats showed a similar trend, however, the results were not statistically significant. All strains tended to show diabetes-induced impairment of dark-adapted b-wave amplitude, but only Sprague Dawley and Lewis strains had a significant reduction in latency. All strains showed significant tactile allodynia in peripheral nerves.

**Conclusions:**

At the durations studied, Lewis rats showed accelerated loss of both retinal capillaries and ganglion cells in diabetes, whereas diabetic Wistar rats showed degeneration of the capillaries without significant neurodegeneration, and Sprague Dawley rats showed neither lesion. Identification of strains that develop retinal lesions at different rates should be of value in investigating the pathogenesis of retinopathy.

## Introduction

Hyperglycemia is accepted as a major determinant of susceptibility to diabetic retinopathy, neuropathy, and nephropathy. Nevertheless, some patients in poor glycemic control have escaped these complications and some patients in good glycemic control have developed retinopathy [1]. Evidence indicates that the severity of diabetic retinopathy is influenced by familial (possibly genetic) factors in Type 1 [1] and Type 2 diabetes [2]. Moreover, a possible role of genetics in the development of retinopathy has been suggested by monozygotic twin pairs showing greater concordance for severity of retinopathy than dizygotic twin pairs with Type 2 diabetes [3], a higher prevalence of background retinopathy in non-Hispanic than in Hispanic whites with Type 2 diabetes [4], and differences in rates of nonproliferative and proliferative retinopathy in non-Ashkenazi versus Ashkenazi Jews with Type 1 diabetes [5].

Efforts to identify genetic contributors to retinopathy have primarily involved the candidate gene approach. The severity of retinopathy has been found to be associated with gene polymorphisms of the aldose reductase pathway [6–17], the renin-angiotensin system [18–20], and the human leukocyte antigen (HLA) system [21–23]. However, these loci have shown only modest associations with retinopathy and findings generally have not been replicated in other population groups. Thus, the role of genetics in the pathogenesis of diabetic retinopathy has remained difficult to assess [24].

Animal models have been used to investigate genetic factors important in the development of ocular vascular permeability [25] and neovascularization [26–28]. Although diabetic rats have not been found to develop the advanced stages of diabetic retinopathy seen in humans, they can reproduce lesions of early diabetic retinopathy, including structural (capillary degeneration, pericyte loss, ganglion cell loss) and functional (electroretinography [ERG] abnormalities) abnormalities [29–31]. Strain differences in the rate of development of kidney disease have provided insight into the pathogenesis of chronic kidney disease [32] and kidney disease in diabetes [33], where susceptibility to glomerulosclerosis in diabetes was judged to be at least in part inherited, with hyperglycemia serving principally as a trigger in the development of the nephropathy.

We sought to investigate potential differences in the rate at which early stages of diabetic retinopathy and sensory hyperaesthesia develop in several rat strains. Previous studies conducted using different rat strains have detected retinopathy lesions at different durations of diabetes [34–40]. These differences might simply have been due to differences in study duration or in the severity of hyperglycemia between studies, but strain-dependent differences in the susceptibility to diabetic retinopathy was also a possibility. Thus, we conducted a side-by-side comparison of retinopathy development in three rat strains (Sprague Dawley, Lewis, and Wistar). For comparison, we also evaluated the strains with respect to the rate at which diabetes-induced tactile allodynia developed.

## Methods

### Animal models

Sprague Dawley, Lewis, and Wistar rats (200 g, male) were purchased from Harlan Laboratories (Indianapolis, IN) and kept in ventilated microisolator units. Insulin-deficient diabetes was induced with streptozotocin (55 mg/kg BW) after an overnight fast. All experiments followed the guidelines set forth by the Association for Research in Vision and Ophthalmology Resolution on Treatment of Animals in Research. The experiment did not begin until two weeks after streptozotocin was administered to ensure all animals were satisfactorily diabetic. All animals were fed Teklad 7004 (Harlan Teklad, Indianapolis, IN). If any animals lost weight at any time during the study, they were given insulin (initially two units Neutral Protamine Hagedorn insulin twice per week), with more given later if necessary to achieve slow weight gain without preventing hyperglycemia and glucosuria. Thus, diabetic rats were insulin-deficient, but not grossly catabolic. Hyperglycemia was estimated every two to three months by glycated hemoglobin (GHb) assay using a VARIANT kit (Bio-Rad, Hercules, CA) and by repeated blood glucose concentration assays. Diabetic and age-matched nondiabetic controls were sacrificed at two months of diabetes, eight months of diabetes (for histopathology), and four months (for western blots, electroretinograms [ERG], and neurodegeneration). Eight months’ duration was selected for the assessment of histopathology because six to eight months is the earliest we have been able to detect a diabetes-induced increase in degenerate capillaries that achieves statistical significance, thus making it a sensitive time to determine if the other strains developed the pathology faster or slower.

### Western blot analysis

Retinas were sonicated in radioimmunoprecipitation assay (RIPA) buffer (25 mM Tris [pH=7.4], 1 mM EDTA, 150 mM NaCl, 1% NP-40, 0.1% sodium dodecyl sulfate [SDS], 0.5% deoxycholic acid, 1 mM phenylmethylsulfonyl fluoride, 1 μg/ml leupeptin and 1 μg /ml aprotinin). Proteins were fractionated by SDS–PAGE (PAGE), and anti-inducible nitric oxide synthase (iNOS) polyclonal antibody (1:1,000 dilution; Transduction Laboratories, Lexington, KY), cyclooxygenase-2 (COX-2) polyclonal antiserum (1:500 dilution; Cayman Chemical, Ann Arbor, MI), or anti-vascular endothelial growth factor (VEGF) polyclonal antibody (sc-152; Santa Cruz Biotechnology) were administered. After extensive washing, protein bands detected by the antibodies were visualized by enhanced chemiluminescence and evaluated using densitometry. Membranes were then stripped and re-probed with antibody against actin (Sigma, St. Louis, MO) to confirm equal protein loading. One retina from each of six animals per experimental group was analyzed using western blots.

### Isolation of retinal blood vessels

The retinal vasculature was isolated using the trypsin digest method, as previously described [38,41–43]. Briefly, freshly isolated eyes were fixed with 10% neural buffered formalin. Retinas were isolated, washed in water overnight, and then incubated with 3% Difco crude trypsin (BD BioSciences, Sparks, MD) at 37 °C for 1 h. Non-vascular cells were gently bushed away from the vasculature and the isolated vasculature was laid out on glass slides and air-dried for a terminal deoxynucleotidyl transferase dUTP nick end labeling (TUNEL) assay and pathological examination.

### Quantitation of acellular capillaries and pericyte ghosts

Slides were stained with hematoxylin and periodic acid-Schiff, dehydrated and coverslipped. Acellular capillaries were quantified in four to seven field areas in the mid-retina (200× magnification) in a masked manner. Acellular capillaries were identified as capillary-sized vessel tubes having no nuclei anywhere along their length, and were reported per square millimeter of retinal area. Tubes having a diameter <20% of the adjacent capillaries were identified as strands and were not counted as acellular capillaries. Pericyte ghosts were estimated from the prevalence of spaces in the capillary basement membranes from which pericytes had disappeared. At least 1,000 capillary cells in five field areas in the mid-retina (400× magnification) were evaluated in a masked manner, and the number of pericyte ghosts was reported per 1,000 capillary cells. Ghosts on any acellular vessel were excluded.

### Neurodegeneration

Cells in the ganglion cell layer (GCL) were counted as a parameter of diabetes-induced retinal neurodegeneration. Formalin-fixed eyes were embedded in paraffin, sectioned sagittally through the retina (going through the optic nerve), and stained with hematoxylin and eosion (H&E). The number of cells in the GCL was counted per 250 μm retinal length in two areas (mid-retina and posterior retina adjacent to the optic nerve) on both sides of the optic nerve. Results from all four regions were expressed per unit length and averaged.

### Electroretinogram (ERG)

Measurements were made as described previously [44] for five or six animals per group. Rats diabetic for four months (and nondiabetic controls) were dark-adapted overnight, anesthetized intraperitoneally with ketamine (4 mg/100 g BW) and xylazine (1 mg/100 g BW), and placed on a heating pad during the recording session. Pupils were dilated with 1% tropicamide, 1% cyclopentalate hydrochloride, and 2.5% phenylephrine hydrochloride. Recordings were made using a stainless steel wire loop that contacted the corneal surface through a thin layer of 1% methylcellulose. Needle electrodes placed in the tail and cheek served as ground and reference electrodes, respectively. Responses were amplified (1–1,000 Hz), averaged, and stored on an LKC (UTAS) signal averaging system.

A dark-adapted intensity-response series was recorded using a series of Ganzfeld flashes with intensities ranging from –4.2 to 0.5 log cd sec/m^2^ to obtain rod-mediated retinal responses. Cone-mediated responses were obtained to light stimuli after 7 min light adaptation in which the animals were exposed to a steady rod-desensitizing background light of 0.8 log cd/m^2^ presented in the Ganzfeld bowl. During steady rod desensitization, cone-mediated responses to a series of flash intensities were elicited (−0.22 to 0.52 log cd sec/m^2^). The average response to 25 flashes was calculated for each intensity. The amplitude and latency of individual ERG waveform components (the a- and b-waves) were measured conventionally. For the a-wave, amplitude was measured from the pre-stimulus baseline to the trough. For the b-wave, amplitude was measured from the negative trough of the a-wave to the b-wave peak. Latency, or time-to-peak, was measured from stimulus onset to the a-wave trough and the b-wave peak.

### Tactile allodynia

This test was performed as described by Chaplan et al. [45,46], as previously reported [47], at two months of diabetes. Briefly, animals were placed in cages with wire-mesh bottoms and the plantar surface of the hind paws were poked through the mesh using Von Frey filaments (Stoelting, Chicago, IL) to determine the 50% mechanical threshold for the animal to lift or lick its paw. Lack of a response after 5 s prompted the use of another filament of different stiffness, and results from both hind paws for each animal were averaged together. All measurements were made by an investigator who was unaware of the treatment group that individual animals were in.

### Statistical analysis

Electroretinography results across different light intensities and at individual intensities were assessed by the Repeated Measures test and *t*-tests. All other measures were analyzed using ANOVA (ANOVA) and Fisher post-hoc tests. Differences were considered statistically significant when p values were <0.05.

## Results

Glycated hemoglobin and blood glucose in diabetic rats were significantly greater than in nondiabetic controls in each of the three strains studied. The severity of diabetes-induced hyperglycemia was similar among all three strains (Table 1). During the eight-month experiment, GHb was higher in Sprague Dawley and Wistar rats than in Lewis rats, but average fasting blood glucose over the course of the experiment did not differ among groups. Diabetic rats were treated with insulin so they did not lose weight, but with amounts that were insufficient to maintain growth at the rate shown by nondiabetic rats. Thus, bodyweights of all groups of diabetic rats remained significantly less than those of nondiabetic control rats (due to impaired growth, not weight loss). Amounts of insulin injected tended to differ among groups, but the differences were not statistically significant.

**Table 1:** Study of nondiabetic and diabetic parameters in three strains.

**Strain and group**	**n**	GHb (%)*	Fasting blood glucose* (mM)	**Insulin injected (U/kgBW/month)**	**Final bodyweight (g)**
**Sprague Dawley**					
Nondiabetic	≥8	3.0±0.2	2.9±0.4	-	656±27
Diabetic	≥8	11.2±1.7	17.2±3.2	35.7±42.9	309±63
**Lewis**					
Nondiabetic	≥8	2.9±0.6	2.9±0.2	-	555±48
Diabetic	≥8	10.1±1.3	13.3±2.7	22.5±24.9	307±25
**Wistar**					
Nondiabetic	≥8	4.2±1.8	2.8±0.4	-	524±134
Diabetic	≥8	11.4±1.5	13.8±6.0	6.8±5.0	325±91

*Average over the 8 month duration of study. GHb represents Glycated hemoglobin.

**Figure 1:**
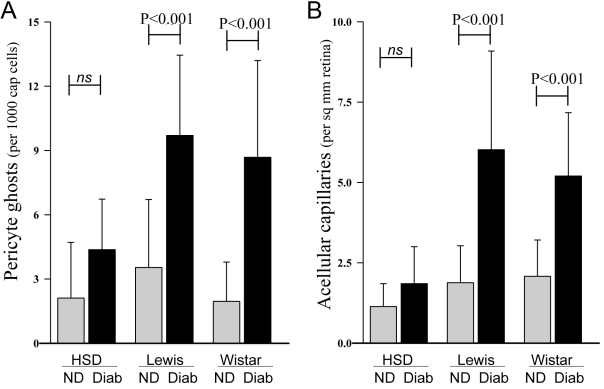
Comparison of capillary degeneration among different rat strains. Diabetes-induced degeneration of retinal capillaries (**A**) and pericyte loss (**B**) occurred more slowly in Sprague Dawley rats than in Lewis or Wistar rats diabetic (Diab) for eight months or in age-matched nondiabetic (ND) controls. Both lesions were counted in a masked fashion on trypsin-digested microvessel preparations. All groups contained more than eight animals. Mean±SEM.

Eight months of diabetes did not induce degeneration of retinal capillaries equally among the three strains tested. In Lewis and Wistar rats, diabetes of eight months’ duration caused a significant increase in the number of degenerate (acellular) capillaries and pericyte ghosts compared to nondiabetic controls (approximately threefold greater than controls, each p<0.001). In contrast, the same duration of diabetes did not cause a significant increase in the number of degenerate capillaries or pericyte ghosts in Sprague Dawley rats (Figure 1).

**Figure 2:**
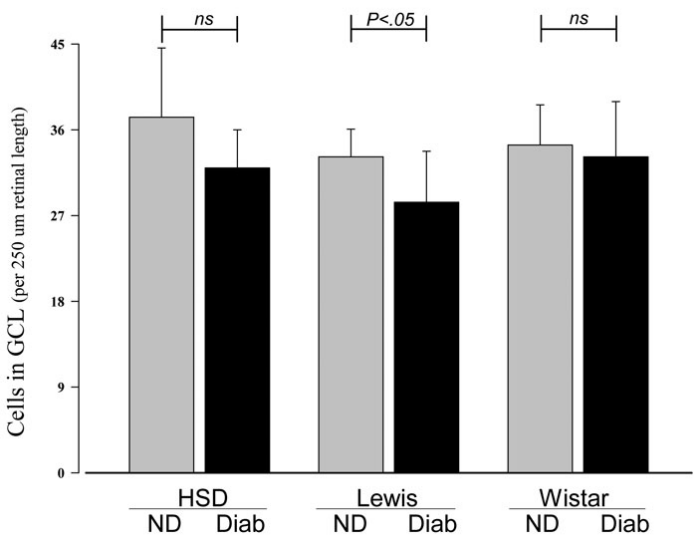
Diabetes of four months’ duration caused a significant decrease in the number of cells in the GCL of the retina in Lewis rats, but not Wistar rats. Sprague Dawley diabetic rats tended to show fewer cells in the GCL compared to nondiabetic controls, but the results were not statistically significant. All groups contained more than eight animals. Mean±SEM.

**Figure 3:**
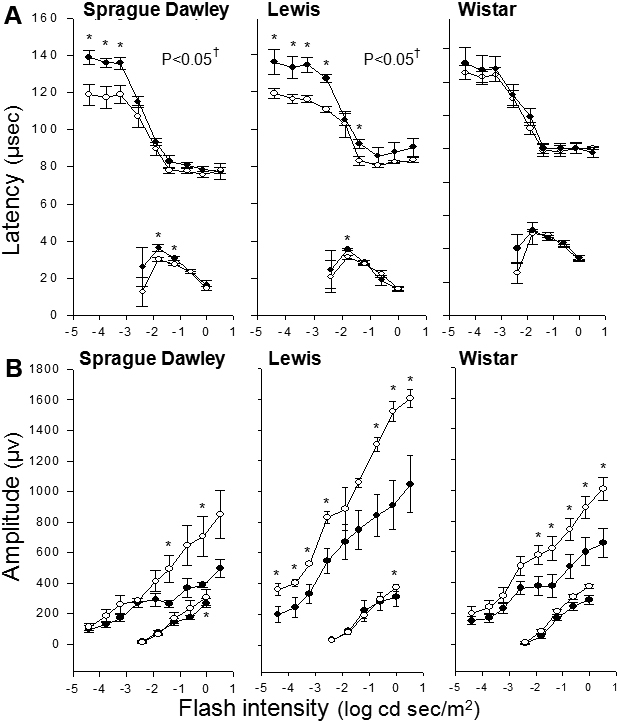
Diabetes of 4 months duration reduced the latency of rod-mediated b-waves in Sprague Dawley and Lewis strains, but not Wistar rats. Electroretinogram assessed responses to a bright flash in diabetic rats (solid circles) or age-matched nondiabetic controls (hollow circles). Peak amplitudes of rod mediated a- and b-wave latency (**A**) and amplitude (**B**) are graphed as a function of strobe flash intensity. Amplitude tended to be subnormal in all 3 diabetic strains, but results achieved statistical significance mainly at higher intensities. Group sizes were 5 for all measurements. †p<0.05 by repeated measures test. *p<0.05 by *t*-test between nondiabetic and diabetic at specified flash intensity. Mean±SEM.

Nonvascular cells of the retina have also been reported to degenerate in diabetes. At four months of diabetes, the number of cells in the GCL was counted in retinal cross-sections through the optic nerve. As summarized in Figure 2, only Lewis rats showed a significant reduction in the number of cells in the GCL compared to nondiabetic controls (p<0.05). The statistical significance of this reduction in the Lewis strain was due to changes in the posterior retina only; none of the strains showed a statistically significant reduction in numbers of cells in the GCL in the mid-retina region (not shown). To determine if strain differences in the rate of ganglion cell loss influenced retinal function, we also measured retinal function via light- and dark-adapted ERG in diabetic and nondiabetic animals of all three strains. Lewis rats showed greater changes in amplitude in response to light than did other strains. Diabetes of four months’ duration reduced the latency of rod-mediated b-waves in Sprague Dawley and Lewis strains (p<0.05; Figure 3). Amplitudes of the dark-adapted b-waves tended to be subnormal in diabetics of all three strains, but the results were not statistically significant. Comparisons at individual light intensities (by *t*-test) indicated that rod-mediated b-wave amplitudes in diabetics were significantly different from normal only at higher intensities for all three strains. The only cone-mediated b-wave process that was significantly altered by diabetes was amplitude, only at the highest intensity, in Sprague Dawley and Lewis strains (not shown).

**Figure 4:**
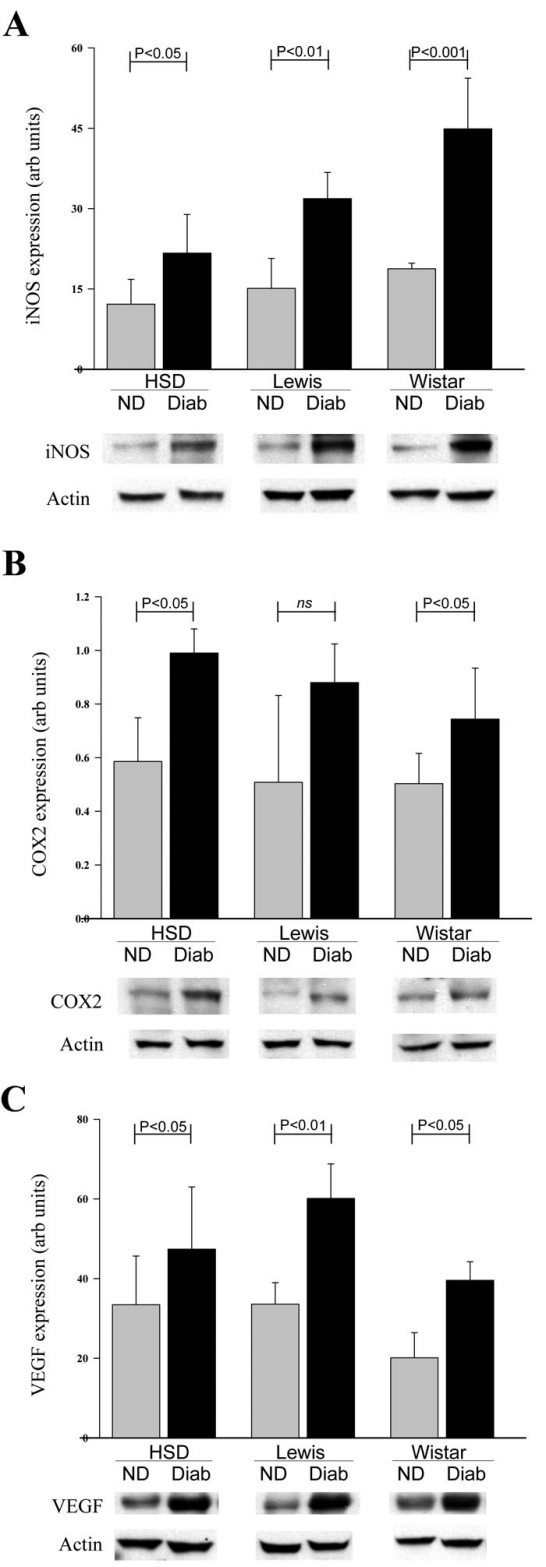
Comparison of diabetes-induced changes in inflammatory molecules among rat strains. Diabetes of four months’ duration increased retinal expression of (**A**) inducible nitric oxide synthase (iNOS), (**B**) cyclooxygenase 2 (COX2), and (**C**) vascular endothelial growth factor (VEGF) in Sprague Dawley, Lewis, and Wistar rats. All diabetes-induced increases were statistically significant, except that COX2 expression only tended to increase in Lewis rats. Expression data are expressed as a ratio to expression of actin in the same sample. Groups contained six animals per group. Mean±SEM.

Inflammatory processes have been postulated to contribute to the development of diabetic retinopathy [48,49]. In an effort to identify possible biochemical causes of the observed strain differences, we measured several biochemical parameters involved in the inflammatory process. Compared to age-matched nondiabetic controls for each of the strains, diabetes tended to increase expression of iNOS, COX2, and VEGF in all three strains (Figure 4). The diabetes-induced increase in protein expression was statistically significant for all strains with respect to iNOS and VEGF, and was significantly increased in Sprague Dawley and Wistar strains for COX2. Cyclooxygenase 2 expression tended to increase in diabetic Lewis rats, but the increase was not statistically significant at this duration of diabetes.

**Figure 5:**
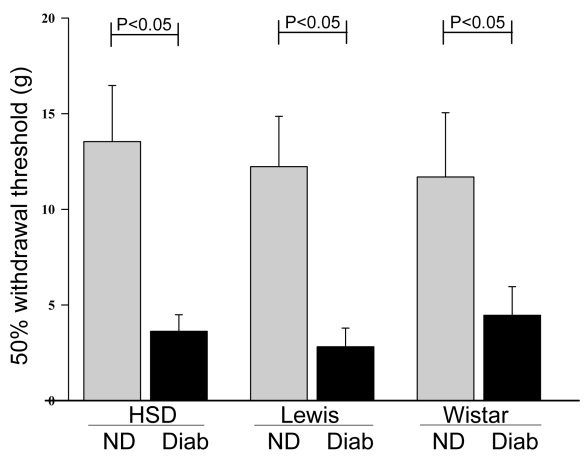
Diabetes also affects the peripheral nervous system. Two months of diabetes results in hypersensitivity to light touch (tactile allodynia) in the hind paws of all three rat strains studied (Sprague Dawley, Lewis, and Wistar rats). Groups contained at least six animals per group. Mean±SEM.

Rats diabetic for two months detected peripheral sensory stimulation at significantly lower pressures than nondiabetic animals in all three rat strains (Figure 5; p<0.01). Despite the difference among strains in the rate of development of retinal lesions, little or no difference among strains was noted with regard to the severity of diabetes-induced tactile allodynia.

## Discussion

In the present study, three different rat strains were compared with respect to their susceptibility to develop early-stage diabetic retinopathy. These vascular and neural abnormalities also develop in diabetic humans, and evidence suggests that they contribute to the later development of clinically meaningful visual impairment in diabetic patients. We found that diabetes-induced degeneration of retinal capillaries occurred relatively faster in Lewis and Wistar strains than in Sprague Dawley rats. Diabetes-induced degeneration of retinal ganglion cells likewise developed at different rates among the strains, but the results were not the same as for capillary degeneration. Only Lewis rats showed a significant loss of retinal ganglion cells within four months of diabetes. Importantly, these strain differences in susceptibility or resistance to retinal vascular and neuronal lesions in diabetes seem not to be attributable to differences in glycemia.

Differences between rat strains have also been reported for other parameters, including susceptibility to blood-retinal barrier breakdown in diabetes. Diabetic Brown Norway rats developed sustained vascular hyperpermeability in the retina over a period of 16 weeks, whereas diabetic Sprague Dawley rats showed only transient hyperpermeability immediately following diabetes onset [25]. The strain difference in vascular leakage between diabetic rats of the two strains was partially ascribed to different VEGF expression and VEGF signaling in these strains. In our studies, VEGF expression was significantly increased in all three strains, and thus could not be clearly correlated with the rate at which the strains developed vascular pathology in diabetes. Sprague Dawley and Lewis strains have also been shown to respond differently in studies of oxygen-induced retinopathy, hyperoxia causing significantly larger areas of retinal avascularity in Lewis rats compared to Sprague Dawley rats [50]. Differences between these two strains have also been reported with respect to neuropathy and pain, immunoglobulin A (IgA) nephropathy, and inflammation [51–60]. Previous investigators have reported a 10%–15% loss of thickness of the inner plexiform layer and subnormal thickness of the photoreceptor layer one month after the induction of diabetes in Sprague Dawley and Brown Norway rats [61].

At the durations of diabetes studied, Lewis rats showed accelerated loss of both capillaries and ganglion cells in diabetes, whereas diabetic Wistar rats showed degeneration of the capillaries without significant neurodegeneration and Sprague Dawley rats showed neither. Although degeneration of retinal capillaries and ganglion cells in diabetes developed slower in Sprague Dawley rats than in the other strains tested, both lesions nevertheless do eventually develop in Sprague Dawley rats at longer durations of diabetes [35,38,39,62]. Thus, these studies apparently demonstrate genetic differences among the strains that influence the rate at which retinal cells degenerate. However, whether the final extent of degeneration likewise differs has yet to be determined.

Retinal function was also disturbed in diabetic rats of all three strains, but differences among the strains were again apparent. In contrast to findings of vascular and neuronal degeneration, diabetics from the Sprague Dawley and Lewis strains showed more abnormalities in rod-mediated retinal function than did those from the Wistar strain at the duration of diabetes studied. Whether development of these functional defects are related to or predict the rate at which vascular and neural cells degenerate in diabetes cannot be learned from the present study. We previously [63] found that Lewis rats diabetic for a longer duration (nine months) had different responses than reported herein (latency response was not significantly altered in diabetes), raising the possibility that the disturbances of retinal function might change with increasing duration of diabetes.

Differences in the severity of hyperglycemia or insulin deficiency between strains obviously might influence the development rate of pathology. The observed differences in GHb between the diabetics of each strain seem modest, but even if one assumes that those differences are important, the strain with the apparently highest hyperglycemia (Sprague Dawley) developed the pathology the slowest, and the strain with the apparently lower hyperglycemia (Lewis) developed the pathology fastest. The amount of insulin administered to maintain that level of hyperglycemia also seemed different between strains. Insulin can directly affect retinal cells [64], but the amounts of insulin administered to the strains developing capillary degeneration fastest (Lewis) and slowest (Sprague Dawley) were not significantly different. Thus, we conclude that differences in hyperglycemia and the amount of insulin administered are not likely to account for the observed differences in histopathological development in these animals.

Retinas from diabetic animals exhibit biochemical and physiologic abnormalities consistent with inflammation [43,63,65–69], and we and others have postulated that inflammatory processes play an important role in the pathogenesis of diabetic retinopathy [48,49]. Intravitreal administration of the proinflammatory growth factor VEGF has been observed to cause diabetic-like capillary lesions, including capillary leakage and closure, microaneurysms, and intraretinal hemorrhages [70–72]. The extent to which diabetes induced retinal levels of the proinflammatory molecules iNOS, COX2, and VEGF, however, was similar among the three strains studied. Thus, expression levels of at least these inflammatory molecules in the retina seems not to account for the observed strain differences in the rates at which diabetes-induced retinal cell degeneration develops, at least at the duration of diabetes when they were measured. The amount of these proteins expressed might have been quite different at different durations of diabetes. Although inhibition of inflammatory-like processes inhibit development of the retinopathy in animal models [39,42,43,48,63,73–77], other factors, including perhaps blood pressure, lipid levels, and genetic differences, likely also contribute and perhaps are even more important than inflammatory changes in determining the rate at which the retinopathy develops.

Diabetic neuropathy is one of the most common complications of diabetes and includes clinical symptoms such as tactile allodynia (nociceptive responses to normally innocuous stimuli), hyperalgesia (augmented pain response to painful stimuli), and spontaneous pain. Mechanical allodynia, assessed using Von Frey filaments, develops rapidly in diabetic rats and can be inhibited by a variety of therapies, including good glycemic control [46,78]. Although diabetes appreciably enhanced sensitivity to sensory stimuli in our studies, we did not observe differences in the rate at which this complication developed among the different strains tested.

Genetic differences between animal strains or genetically different populations have been used to identify genes responsible for pathology in other diseases (phenotype-to-gene approach). The genetic factors underlying the phenotypic variation among animal strains or populations have been mapped by quantitative trait locus (QTL) analysis, and genes for several QTLs (such as obesity, insulin resistance, taste, susceptibility to cytomegalovirus, and hypertension) have been identified [79–84]. This or similar approaches might be used successfully to identify genes that contribute to susceptibility to diabetic retinopathy. The data we report herein provide essential information for designing such experiments.
